# Machine-Learning-Based Indoor Localization under Shadowing Condition for P-NOMA VLC Systems

**DOI:** 10.3390/s23115319

**Published:** 2023-06-03

**Authors:** Affan Affan, Hafiz M. Asif, Naser Tarhuni

**Affiliations:** 1 Department of Electrical and Computer Engineering, University of Louisville, Louisville, KY 40292, USA; affan.affan@louisville.edu; 2 Department of Electrical and Computer Engineering, Sultan Qaboos University, Al-Khoud, Muscat 123, Oman; tarhuni@squ.edu.om

**Keywords:** visible light communication, machine learning, SIC, NOMA, localization, shadowing

## Abstract

The localization of agents for collaborative tasks is crucial to maintain the quality of the communication link for successful data transmission between the base station and agents. Power-domain Non-Orthogonal Multiple Access (P-NOMA) is an emerging multiplexing technique that enables the base station to accumulate signals for different agents using the same time-frequency channel. The environment information such as distance from the base station is required at the base station to calculate communication channel gains and allocate suitable signal power to each agent. The accurate estimate of the position for power allocation of P-NOMA in a dynamic environment is challenging due to the changing location of the end-agent and shadowing. In this paper, we take advantage of the two-way Visible Light Communication (VLC) link to (1) estimate the position of the end-agent in a real-time indoor environment based on the signal power received at the base station using machine learning algorithms and (2) allocate resources using the Simplified Gain Ratio Power Allocation (S-GRPA) scheme with the look-up table method. In addition, we use the Euclidean Distance Matrix (EDM) to estimate the location of the end-agent whose signal was lost due to shadowing. The simulation results show that the machine learning algorithm is able to provide an accuracy of 0.19 m and allocate power to the agent.

## 1. Introduction

Visible Light Communication (VLC) systems have been the focus of research and development for many years. Numerous VLC products have been developed and are commercially available such as Light Fidelity (LiFi) [1]. The basic components of VLC systems include Light Emitting Diodes (LEDs) and Laser Diodes (LDs) as transmitters and Photo-Detectors (PDs) as the receivers [2,3]. At the transmitter end, the information bits are converted into electrical signals to drive LEDs, while at the receiver end, the photons are received by PDs and converted back to information bits. VLC systems use the visible light spectrum instead of the Radio Frequency (RF) spectrum. The wide range of the visible light spectrum, 380–780 nm, is one of the main advantages of VLC systems over RF systems. VLC systems also provide a higher data rate [4,5]. VLC systems may not replace RF systems completely; however, these systems can be used as hybrid technologies to improve communication quality for different applications and environments [6].

VLC systems are used for both data communication and illumination. This leads to a wide range of applications [7]. Indoor environments are prime examples of the application of VLC systems due to the availability of pre-installed infrastructure for the transmitters [8]. In [9], the authors proposed an indoor VLC system based on On–Off Keying (OOK) using four devices equipped with 3600 LEDs (60 × 60), which were installed in a room of dimensions 5.0 m × 5.0 m × 3.0 m. The authors of [9] concluded that the system can satisfy communication requirements, such as the high data rate of 10 Gbps can be achieved using LEDs with OOK and intersymbol interference is dependent on the data rate and the FOV of the receiver in the indoor environment. In [10], a data rate of about 200 Mbps was achieved using blue LEDs for an indoor environment. The laser-based white-light-emitting Surface Mount Device (SMD) platform combined with blue LEDs is proposed in [11], and the proposed framework achieved a data rate of about 20 Gbps with 10–100 times more brightness as compared to the conventional light bulbs.

Indoor positioning methods using VLC technologies have been reported in numerous studies. The features such as Received Signal Strength (RSS), Time of Arrival (ToA), and Angle of Arrival (AoA) are the most prominent when designing an indoor positioning system. The first, high-precision VLC-based indoor positioning system was proposed in [12]. The proposed algorithm uses RSS measurement to obtain the user/object location with an accuracy of 0.4 m. In [13], AoA is used as a feature to find the user/object location with an accuracy of 0.1 m. The proposed framework used image sensors instead of PDs. The hybrid approach using a combination of RSS and AoA for improved communication and localization is proposed in [14,15]. In the aspect of cost-effectiveness, Ref. [16] proposed a two-stage framework. First, they presented a dedicated analog sensor that is capable of being directly plugged into the microphone input of a computer or a mobile device such as a smartphone. The signal pattern, as well as the signal strength of a beacon, can both be decoded by it. Second, to decode the signal pattern, the use of rolling shutter cameras is proposed. This offers a viable answer to the problem of localizing hand-held devices that contain cameras.

Artificial Intelligence (AI) methods have been widely developed for the application of indoor positioning mainly due to high accuracy and easy deployment [17,18]. The machine learning-based classifiers are compared for localization in an indoor environment in [19]. It has been reported that the k-nearest neighbor (k-NN) algorithm performed the best among all other classifiers. It has been reported in [20] that support vector machine (SVM) outperformed logistic regression using the fingerprinting method for Bluetooth signals in an indoor environment. In [21], Principle Component Analysis (PCA) is used to improve the performance of SVM, K-NN, and random forest classifiers for indoor positioning. In [22], it is reported that the random forest classifier outperforms K-NN for WiFi-based indoor positioning. In [23], an enhance random forest algorithm is proposed for indoor positioning in real-world settings. The proposed machine learning algorithms have proven to be efficient; however, a comprehensive analysis for a multi-agent system that also considers the signal loss due to obstacles is missing a link. Therefore, firstly this paper compares the performance of SVM and Random Forest Regression (RFR) for VLC-based indoor positioning and used Euclidian Distance Matrix (EDM) to find the location of the agent in the scenario of signal loss due to obstacles. Secondly, we complete the framework with adaptive resource allocation for multi-agent systems based on obtained positions.

VLC technologies have been used for a variety of autonomous systems such as vehicles, autonomous ground robots, and fixed robotic industrial grippers. In [24], an in-hospital transportation robot called HOSPI is developed and VLC technology is used to improve navigation and localization in addition to other navigation sensors for better autonomous control of the robot. The study explores the localization and autonomy using experimental and actual results in an actual hospital. In [25], the multi-frequency method with RSS is used to measure the distance of the robot from each LED that is installed above the robot in a plane that is parallel to the plane of the robot base in order to accomplish precise indoor positioning. This is accomplished by installing the LEDs in a plane that is perpendicular to the surface of the robot base. A multi-frequency technique is one in which each LED transmits its location ID at a frequency that is distinct from the others. In [26], VLC technology in addition to the Extended Kalman filter has been used for communication and localization in underwater robots for nuclear reactor inspection. VLC-based systems have proven to be efficient in the area of positioning for multiple applications; however, these proposed studies often require high-cost, sophisticated setups for high accuracy. Therefore, in this research, a system is described that makes use of the modulated light signal both as a medium through which data can be sent and as a reference upon which to base the positioning of a mobile robot/agent. Both of these functions are carried out within the same framework.

Moreover, the underlying modulation and multiplexing techniques for VLC systems have constantly evolved to meet the needs of more complicated and dynamic environments. Many different approaches, such as Orthogonal Frequency Division Multiple Access (OFDMA), Time Division Multiple Access (TDMA), and Code Division Multiple Access (CDMA), have been suggested in the research literature as methods for implementing multiple access in high bit rate VLC systems [27,28,29,30,31,32,33,34]. MIMO-OFDM is utilized for multiuser VLC systems in [35], and this is accomplished by giving each user their own unique carrier. In the same vein, CDMA is used in conjunction with OFDM to support multiple-user communication rather than sending a single carrier to each user. Similarly, Non-Orthogonal Multiple Access (NOMA) methods have been widely proposed in VLC systems to increase the number of users without compromising on performance [36]. In the power domain NOMA, the data of different agents are accumulated together using different power factors for each agent, and Successive Interference Cancellation (SIC) is used to retrieve the data at the receiver end. However, the resource allocation for the power domain NOMA is highly affected by the channel and environment.

The resource allocation in NOMA has been a focus of research for many years [37]. In [38], the subject of energy-efficient user planning and power optimization in NOMA wireless links is investigated to understand the trade-off that exists between the data rate effectiveness and the amount of energy that is consumed by NOMA. For the downlink NOMA heterogeneous network, energy-efficient user planning and power distribution techniques are presented for both perfect and imperfect Channel State Information (CSI), respectively. In [39], the neural network-based resource allocation method is proposed for mobile users with mutual interference management. This research work also provides priorities and rate demand-based user scheduling methods to coordinate the access of heterogeneous users with limited radio resources. In [40], a low complexity power allocation scheme for NOMA-based indoor VLC systems, which is called the Simplified Gain Ratio Power Allocation (S-GRPA) scheme, is proposed. The CSI used for power allocation in NOMA is obtained through the look-up table method rather than calculation. In this research work, the location of the agent is received at the base station by a separate low-energy communication link. This approach suffers the loss of the signal-carrying position information. Inspired by this approach, we proposed to use machine learning algorithms to find the location of the agent for CSI for better dynamic resource allocation in NOMA for a collaborative indoor environment instead of the separate communication link. Additionally, to avoid the loss of agent locations due to shadowing or obstacles, we used EDM to obtain the location of agents using mutual distances of agents in the network.

In this research work, we use machine learning algorithms such as RFR and SVM with minimum features of RSS and AoA for an indoor positioning framework using VLC technologies. The positioning algorithm is combined with the S-GRPA scheme for the adaptive resource allocation in a P-NOMA-based multi-agent communication network. Furthermore, we use the Euclidean Distance Matrix (EDM) to estimate the position of the agent whose signal was lost due to shadowing. The simulation results show that RFR-based positioning algorithm is able to provide an accuracy of 0.19 m and allocate power to the agent.

The rest of the article is structured as follows. Section 2.1 discusses the VLC channel followed by a mathematical discussion on the S-GRPA for NOMA in Section 2.2. Section 2.3 and Section 2.4 discusses Random Forest Regression (RFR) and Support Vector Machine (SVM) algorithms. Section 2.5 discusses the Euclidean Distance Matrix (EDM) to obtain an agent’s location in the event of signal loss due to shadowing and obstacles. Section 3 presents results for indoor positioning and the bit-error-rate (BER) for NOMA-based VLC systems. The article is finally wrapped up with concluding remarks.

## 2. System Design

**Figure 1:**
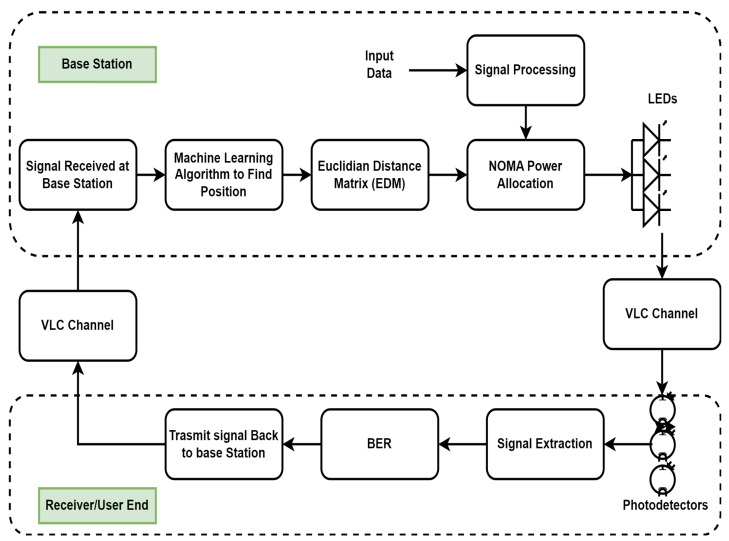
Block diagram for complete NOMA-based VLC system using S-GRPA and indoor positioning.

In this research work, a complete framework for resource allocation for power domain NOMA using S-GRPA, in the indoor environment for collaborative tasks, is proposed using visible light communication systems as shown in Figure 1. For indoor positioning, we have compared the RFR and the SVM algorithms with the Euclidean Distance Matrix (EDM). The complete system block diagram is shown in Figure 1. The next section discusses the S-GRPA for NOMA with the VLC channel model.

### 2.1. Visible Light Communication (VLC) Channel

**Figure 2:**
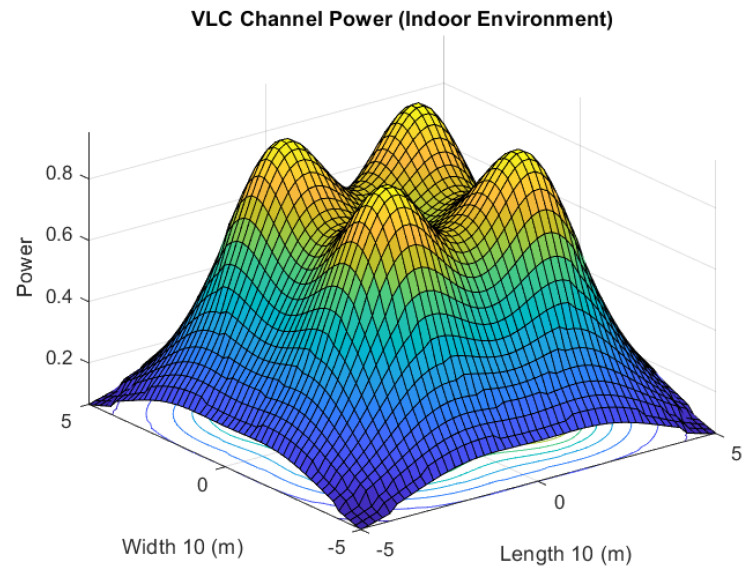
Three-dimensional view of relative power distribution of VLC channel in an indoor environment with four transmitting locations.

LEDs, the transmitting devices in VLC, serve dual tasks by providing both light and data transmission. As can be seen in Figure 2, the responsiveness of the VLC channel in an indoor setting is heavily dependent on the illumination intensity and the transmission power. The illumination intensity at a point in the Cartesian plane is given as follows [41]:

(1) $$I\left(x,y\right)=\frac{I\left(0\right)cos^{m_{l}}\left(\theta \right)}{d^{2}cos\left(\psi \right)},$$

where I(0) is the intensity of the central light source, $m_{l}$, is the order of Lambertian emission, *d* is the separation distance between the LEDs and the PDs, and θ and ψ are the irradiance and incidence angles, respectively. Lambertian emission, $m_{l}$, is described in the following order:

(2) $$m_{l}=\frac{ln(2)}{ln(cos\left(\vartheta_{\frac{1}{2}}\right))},$$

where $\vartheta_{\frac{1}{2}}$ is the angle at half illuminance of an LED. The signal power received at a particular PD is given as follows:

(3) $$P_{r}=P_{t}.\left(\frac{m_{l}+1}{2\pi d^{2}}\right)cos^{m_{l}}\left(\theta \right).T_{s}\left(\psi \right).cos\left(\psi \right),\;0\leq \psi \leq \psi_{con}$$

where $T_{s}\left(\psi \right)$ is the filter transmission, g(ψ) is concentrator gain and $\psi_{con}$ is the field of view of PD. $P_{t}$ is transmitted signal power, and it fades through Line of Sight (LoS) channel gain. The DC gain of the LoS path is given as follows:

(4) $$h_{d}\left(0\right)=\left\{\begin{array}{cc} \left(\frac{m_{l}+1}{2\pi d^{2}}\right)cos^{m_{l}}\left(\theta \right) & \psi \leq \psi_{con}\\ 0 & \psi >\psi_{con} \end{array}\right.$$

The distance, *d*, between the transmitter and receiver is the key factor in the allocation of signal power in NOMA-based communication systems. The next section discusses the mathematical basis for NOMA and resource allocation in this method.

### 2.2. Simplified Gain Ratio Power Allocation (S-GRPA) for NOMA

**Figure 3:**
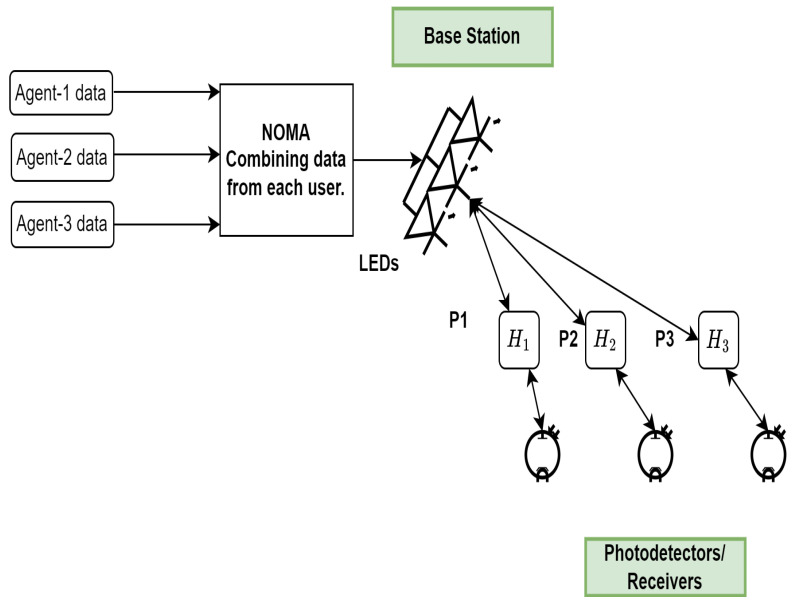
Illustration of NOMA method for VLC systems.

Several agents’ data are combined using the power-domain NOMA, and the data are then separated using SIC. NOMA is free of spectrum spreading and has low degradation SNR performance, in contrast to other multiple access methods such as OFDMA and CDMA, due to the high peak-to-average power ratio, low complex receiver design, and moderate latency [42,43]. Several agents using the same resource simultaneously in NOMA boosts system throughput, but each agent has a distinct power factor. In this study, we employ symbol-level NOMA. Figure 3 illustrates the NOMA framework for three agents. Because of the relative difference in distance from the base station, the agent near the cell edge receives a lot more power than the agent at the center of the cell. The received signal can be expressed as follows [42]:

(5) $$\left[\begin{array}{c} y_{1}\\ y_{2}\\ y_{3} \end{array}\right]=\left[\begin{array}{c} H_{1}\\ H_{2}\\ H_{3} \end{array}\right]\left[\begin{array}{c} p_{1}x_{1}+p_{2}x_{2}+p_{3}x_{3} \end{array}\right]+\left[\begin{array}{c} \eta_{1}\\ \eta_{2}\\ \eta_{3} \end{array}\right].$$

The term $p_{1}x_{1}+p_{2}x_{2}+p_{3}x_{3}$ shows the infusion of data of each user at the base station and is transmitted to each user via the communication channel. These infused data experience different channel effects, such as $H_{1},H_{2},H_{3}$, as shown by Figure 3. The terms $y_{1},y_{2}$, and $y_{3}$ show the data received at each agent after passing through the channel. The information from agent-1 is separated and demodulated as follows at the receiver end:

(6) $${\hat{s}}_{1}=\frac{y_{1}}{p_{1}}.$$

After agent-1’s data have been retrieved, agent 2’s data are recovered by SIC reducing agent-1’s interference in the manner described below:

(7) $$\begin{array}{c} \hat{z}=y_{2}-p_{1}{\hat{s}}_{1}, \end{array}$$

(8) $$\begin{array}{c} {\hat{s}}_{2}=\frac{\hat{z}}{p_{2}}. \end{array}$$

This method can be expanded to a higher number of agents. The power factor, *p*, for each agent depends on its location. The information about the position of agents in a multi-agent system can help to assign appropriate power factors to the agents for the P-NOMA-based VLC system. The power distribution of the VLC system is shown in Figure 2, where it can be seen that overall power distribution can be divided into zones, n=1,2,⋯,N, with radius, $r_{N}$. Here, $r_{n}$ refers to the radius of each zone. Furthermore, zone *N* has the lowest illumination intensity and zone 1 has the highest. The nth region can be defined as follows [40]:

(9) $$\left\{\begin{array}{cc} \left[0,r_{n}\right] & n=1,\\ {}[r_{n-1},r_{n}] & 1<n<N,\\ {}[r_{n-1},r_{e}] & n=N, \end{array}\right.$$

for

(10) $$r_{n}=\sqrt{\frac{nr_{e}^{2}}{N}},n=1,2,\cdots ,N.$$

where $r_{e}$ is the maximum radius of the last zone. The relationship between the kth agent and the k−1th agent in terms of power allocation is described as follows [40]:

(11) $$p_{k}={\left(\frac{H_{1}}{H_{N}}\right)}^{k}\times p_{k-1},$$

Here, the information on channel gain, *H*, is received by the indoor positioning algorithm and stored in the look-up table.

### 2.3. Random Forest Regression Algorithm

The idea of aggregating random decision trees was initially presented in the research papers [44,45,46]. This idea is at the heart of the RFR technique. The following is the formulation of the problem statement for the RFR algorithm:

**Problem 1.**  *Calculate the value of the non-parametric regression function denoted by f(x)=E[y|x]→y=f(x), where p is the dimension of the input vector and $x\in R^{p}$ is the input vector used to estimate the output, y∈R.*

Using the training data set, the RFR method predicts the function $f_{e}\left(x\right)$, which is similar to the actual regression function f(x), and then compares the two. $T_{n}=\left\{\left[|y_{1}|x_{1}\right],\left[|y_{2}|x_{2}\right],\cdots ,\left[|y_{n}|x_{n}\right]\right\}$ is a mathematical expression. The error function can be written as: $E{\left[f_{e}\left(x\right)-f\left(x\right)\right]}^{2}\to 0$, as n→∞. The random forest methodology employs *N* different types of regression trees. The input vector, x, is evaluated on each tree in such a way that the projected value for the $i^{th}$ tree is given as follows [45]:

(12) $$f_{e}\left(x,\theta_{i},T_{n}\right)=\sum_{j\in T_{n}\left(\theta_{i}\right)}^{}\frac{x_{j}\in A_{m}\left(x,\theta_{i},T_{n}\right)y}{M_{m}\left(x,\theta_{i},T_{n}\right)}$$

Here, $\theta_{1},\theta_{2},\cdots ,\theta_{N}$ are the independent random variables associated with each regression tree. $T_{n}\left(\theta_{i}\right)$ are the data points selected prior to the construction of trees. $A_{m}\left(x,\theta_{i},T_{n}\right)$ is the zone containing x and $M_{m}\left(x,\theta_{i},T_{n}\right)$ is the number of data points that fall into $A_{m}\left(x,\theta_{i},T_{n}\right)$.

### 2.4. Support Vector Machine Algorithm

As with the RFR algorithm, the purpose of the Support Vector Machine (SVM) regression algorithm is to map the input x to the desired output *y*. Instead of minimizing the discrepancy between the estimated and true regression functions, $f_{e}$ and *f*, respectively, SVM penalizes the final result so that it can be used in linear regression. y=w.x+b. Let *y* be the true output, and inside an area defined as y±ϵ, SVM will make its predictions. The discrepancy between actual output, *y*, and expected output, *z*, is denoted by the $|z_{i}-y_{i}|<\epsilon$. Two slack variables are added as a penalty if the projected output is outside the region y±ϵ. $\zeta^{+}$ indicates that the projected output lies above the region of y+ϵ, whereas $\zeta^{-}$ indicates that the predicted output lies below the region of y−ϵ. The following is the error function for the SVM regression algorithm [47,48,49,50,51]:

(13) $$min\;C\sum_{i=1}^{L}\left(\zeta_{i}^{+}+\zeta_{i}^{-}\right)+\frac{1}{2}{∥w∥}^{2},$$

subject to:

(14) $$\begin{array}{cc} \; & \zeta^{+}\geq 0,\\ \; & \zeta^{-}\geq 0,\\ \; & y_{i}\leq z_{i}+\epsilon +\zeta^{+},\\ \; & y_{i}\geq z_{i}+\epsilon +\zeta^{-}. \end{array}$$

*C* is the tunable variable that controls the penalty on slack variables and ϵ. To solve (13), the following Lagrange multipliers are introduced [47,51]:

(15) $$\begin{array}{cc} \; & \alpha_{i}^{+}\geq 0,\alpha_{i}^{-}\geq 0,\\ \; & \mu_{i}^{+}\geq 0,\mu_{i}^{-}\geq 0. \end{array}$$

This transforms (13) in the following:

(16) $$\begin{array}{cc} L_{p}= & C\sum_{i=1}^{L}\left(\zeta_{i}^{+}+\zeta_{i}^{-}\right)+\frac{1}{2}{∥w∥}^{2}-\sum_{i=1}^{L}\left(\mu_{i}^{+}\zeta_{i}^{+}+\mu_{i}^{-}\zeta_{i}^{-}\right)\\ \; & -\sum_{i=1}^{L}\alpha_{i}^{+}\left(\epsilon +\zeta_{i}^{+}+z_{i}-y_{i}\right)-\sum_{i=1}^{L}\alpha_{i}^{-}\left(\epsilon +\zeta_{i}^{-}-z_{i}+y_{i}\right). \end{array}$$

Now differentiate (16) to 0, with respect to each variable as below [47,51]:

(17) $$\begin{array}{cc} \; & \frac{\partial L_{p}}{\partial w}=0\Rightarrow w=\sum_{i=1}^{L}\left(\alpha_{i}^{+}-\alpha_{i}^{-}\right)x_{i},\\ \; & \frac{\partial L_{p}}{\partial b}=0\Rightarrow \sum_{i=1}^{L}\left(\alpha_{i}^{+}-\alpha_{i}^{-}\right)=0,\\ \; & \frac{\partial L_{p}}{\partial \zeta_{i}^{+}}=0\Rightarrow C=\alpha_{i}^{+}+\mu_{i}^{+},\\ \; & \frac{\partial L_{p}}{\partial \zeta_{i}^{-}}=0\Rightarrow C=\alpha_{i}^{-}+\mu_{i}^{-}. \end{array}$$

The dual form of the primary $L_{p}$ can be defined as follows:

(18) $$\min_{\alpha^{+},\alpha^{-}}[\begin{array}{c} \sum_{i=1}^{L}\left(\alpha_{i}^{+}-\alpha_{i}^{-}\right)y_{i}-\epsilon \sum_{i=1}^{L}\left(\alpha_{i}^{+}-\alpha_{i}^{-}\right)\\ -\frac{1}{2}\sum_{i,j}^{}\left(\alpha_{i}^{+}-\alpha_{i}^{-}\right)\left(\alpha_{i}^{+}-\alpha_{i}^{-}\right)x_{i}\cdot x_{j} \end{array}],$$

subject to:

(19) $$\begin{array}{cc} \; & 0\leq \alpha_{i}^{+}\leq C,\\ \; & 0\leq \alpha_{i}^{-}\leq C,\\ \; & \sum_{i=1}^{L}\left(\alpha_{i}^{+}-\alpha_{i}^{-}\right)=0. \end{array}$$

The predicted output can be written as [51]:

(20) $$z=\sum_{i=1}^{L}\left(\alpha_{i}^{+}-\alpha_{i}^{-}\right)x_{i}.x+b.$$

This concludes the regression algorithms to obtain the location of the agent based on power received at the receiver. However, in real-world settings, the direct receiver might not be able to receive the agent’s signal due to obstacles or because the signal is highly disrupted due to noise. Therefore, for localization in collaborative indoor environments, the distance geometry problem plays an important role by obtaining the location of the particular agent using the mutual distances of all agents in the network. The next section discusses the distance geometry problem using Euclidian Distance Matrix (EDM).

### 2.5. The Distance Geometry Problem

The VLC systems for indoor environments with dynamic agents often face the loss of signal due to shadowing because of obstacles. To assist the localization in the event of signal loss in a multiagent collaborative environment, the Distance Geometry Problem (DGP) is used in conjunction with the machine learning algorithms discussed in the previous subsection. The objective of the DGP is to find the location of agents using their mutual distances. It is important to mention that agents do not only communicate with the base station during collaborative tasks but also communicate with each other, and this provides extra information about the environment that agents transmit to the base station.

The mutual distances of agents are provided in the form of EDM. The solution to DGP for *N* agents in dimension, *d*, is a matrix, $S\in R^{d\times N}=\left[s_{1},s_{2},\ldots ,s_{N}\right]$, here $s_{i}$ are the coordinates for ith agent. The mutual distance between agents can be referred as $D\in R^{N\times N}=\left[d_{ij}\right]$, here $d_{ij}$ is the distance from jth agent to ith agent. To generate an initial estimate of the matrix $\hat{S}$, multi-dimensional scaling is used. The estimated point cloud, $\hat{S}$ can be mapped to the actual matrix, *S*, using rigid transformation in absolute coordinates. For this transformation, the Procrustes Analysis is performed, which is spectral factorization. For this process, it is assumed that the location of some agents, $N_{a}<N$ is known, and these agents are referred to as anchors. To find the location of missing agents in the network, DGP can be stated as static.

The static DGP consists of three stages to obtain matrix *S* from matrix *D*. The first stage is to obtain a Grammian matrix, $G\in R^{N\times N}=S^{T}S$, as it has a one–one relation with matrix *D*. Grammian matrix *G* can be obtained by following the optimization problem [52,53]:

$$\begin{array}{cccc} \; & \underset{G}{\mathrm{minimize}} & \; & \left|\right|\tilde{D}-W\circ K\left(G\right){\left|\right|}^{2}\\ \; & \mathrm{subject}\;\mathrm{to} & \; & \\ \; & \; & & G⪰0;\;G1=0;\;\mathrm{Rank}(G)\leq d, \end{array}$$

where K(.) is a function, which maps Grammian matrix *G* to matrix *D*. *W* is referred to as a binary mask matrix, the entries with zero values in this matrix show the missing measurement of the received power and ∘ is the Hadamard product. The next stage is to obtain matrix *S* using Grammian matrix *G*. The estimate, $\hat{S}$ can be obtained by the Singular Value Decomposition (SVD) method given that matrices *G* and *S* hold the mathematical relation $G=S^{T}S$.

As mentioned earlier, matrix $\hat{S}$ can be mapped to the actual matrix *S* using rigid transformation along with the information of $N_{a}$ anchors. By denoting the columns of matrix *S* related to anchors as $S_{a}$ and assuming $Y_{a}$ refers to the same columns in $\hat{S}$. Furthermore, to make $S_{a}$ and $Y_{a}$ centered at the origin, assume that $S_{a}$ and $Y_{a}$ are the translated version of *S* and *Y*. The transformation R can be defined as follows:

$$\begin{array}{c} R=\underset{Q:QQ^{T}-I}{\arg\;\min\;}||Q{\bar{S}}_{a}-{\bar{Y}}_{a}{\left|\right|}_{F}^{2} \end{array}$$

The actual matrix *S* can be calculated as follows:

(21) $$S=R\left(\hat{S}-s_{a,c}1^{T}\right)+y_{a,c}1^{T},$$

where $s_{a,c}$ and $y_{a,c}$ refer to the centroids of $S_{a}$ and $Y_{a}$, respectively.

This concludes the mathematical basis for the proposed framework for resource allocation using precise positioning of the agent in a collaborative indoor environment using VLC systems. The next section discusses the performance of indoor positioning using SVM and RFR algorithms, and bit-error-rate (BER) for NOMA-based VLC system.

## 3. Results

In the context of this research, a rectangular indoor environment of a width of 6.3 m, a depth of 2.5 m, and a height of 3 m is being studied. These dimensions represent the area of the environment. Each Cartesian point has a zone that corresponds to it that is represented by a square cell that is 0.3 m^2^ in size. The detailed simulation parameters are shown in Table 1. The purpose of this exercise is to make an estimation of the Cartesian location within the square cell region and use this information to calculate the channel gain for resource allocation for NOMA. The location of agents consists of 2D Cartesian coordinates; however, the environment is three-dimensional. The dimensions 6.3 × 2.5 × 2 m^3^ refer to length × width × height. The base station is considered to be mounted at 2 m height from the ground and agents are moving in 2D plane on the floor.

**Figure 4:**
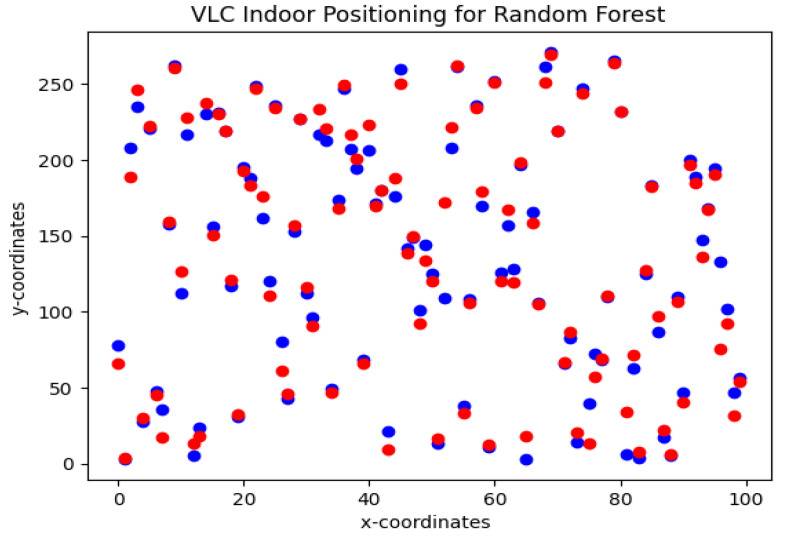
VLC indoor positioning using Random forest regression algorithm. Actual data points (blue), and predicted data points (red).

To obtain the location of agents, the SVM and RFR algorithms are trained using RSS and AoA features at the receiver end. Due to 4×4 Tx-Rx (LED arrays-PDs on agents) configuration for each agent, each feature has a dimension of $R^{4\times 4}$. The dataset is collected in MATLAB using the VLC channel. Each agent has a region of confidence of 0.25 m for its location. Figure 4 shows the estimated and actual Cartesian coordinates of agents. For this simulation, the agent is considered to be dynamic; therefore, different locations are estimated in these simulations for more robustness in the algorithm. The red dots are actual coordinates and the blue dots are estimated values. For RFR, 100 decision trees are used in this simulation. The RFR shows an accuracy of 93.6% with an estimation error of 0.19 ± 0.22 m.

**Figure 5:**
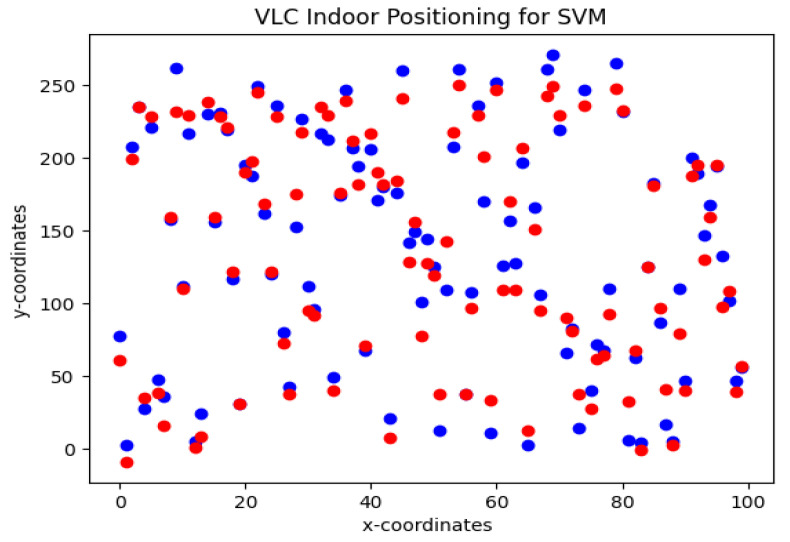
VLC indoor positioning using SVM regression algorithm. Actual data points (blue), and predicted data points (red).

Figure 5 shows the results of the estimated location using the SVM algorithm. For the SVM algorithm, the radial basis function is used to solve the error function. The SVM shows an accuracy of 84% with an estimation error of 0.3±0.2. According to the findings of the statistical analysis, the mean error for the SVM regression method is higher than the mean error for the random forest regression technique. Therefore, RFR is used as a positioning algorithm in conjunction with S-GRPA for power allocation in a NOMA-based multi-agent VLC system.

Table 2 shows the comparison of the proposed RFR and SVM algorithm for VLC-based indoor positioning with other state-of-the-art algorithms. In this table, the Epsilon, Plugo, and Luxapose are algorithms for indoor positioning using VLC technology. The WiDeep is the Wifi-based deep learning algorithm for indoor positioning. It can be seen that RFR with minimum features provides better accuracy than Epsilon, Plugo and WiDeep, and SVM, However, Luxapose is still better than the other algorithms due to more sophisticated hardware and a higher number of features. Therefore, it can be deduced that with minimum features, RFR provides better accuracy and easy deployment.

**Figure 6:**
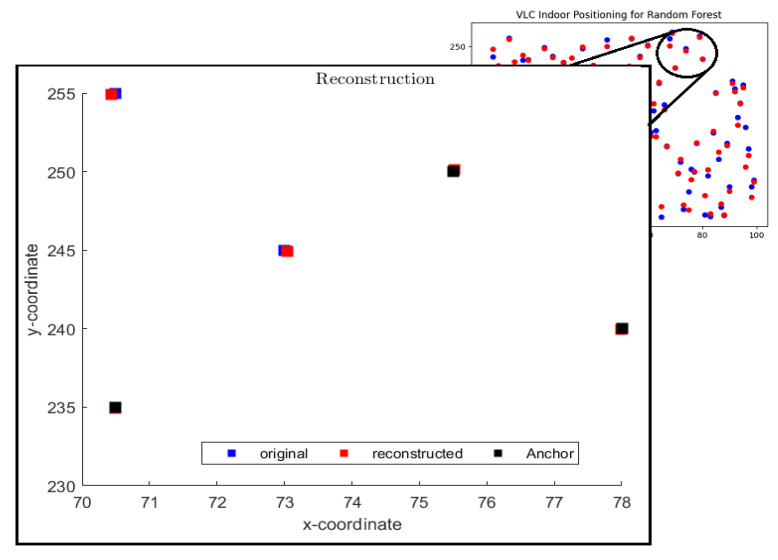
VLC indoor positioning using EDM with RFR, when agent signal is not received at the base due to obstacle. Actual data points (blue), predicted data points using EDM (red), and predicted data points using RFR (black) called anchors.

Figure 6 shows a scenario of five agents where the signal of two agents is not received at the base station due to obstacle/shadowing. In this scenario, the location of the other three agents is predicted by the RFR algorithm and used as anchors for EDM as shown by black data points. The red square boxes in Figure 6 show the estimated position of two remaining agents using EDM. It can be seen that EDM with RFR predicted the locations precisely. Here, the predicted position of anchors (black boxes) is considered to be accurate during the process of EDM.

**Figure 7:**
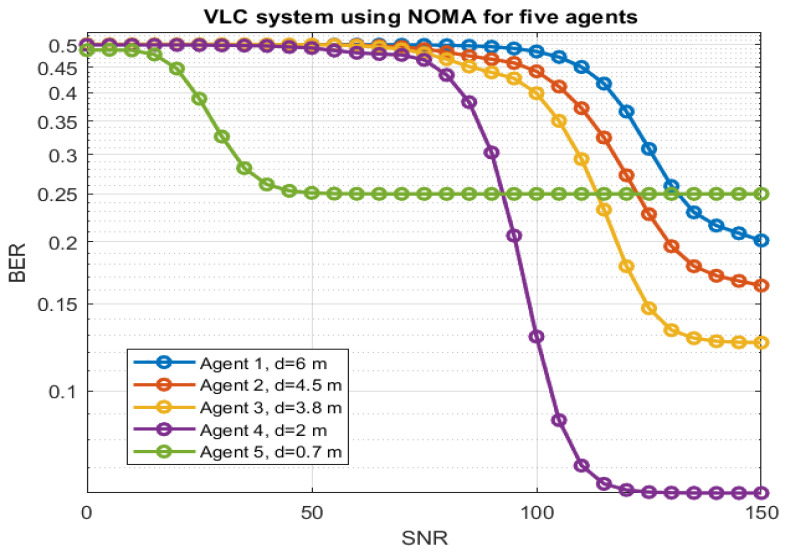
BER results of five agents for NOMA-based LoS VLC system.

By combining the RFR algorithm with S-GRPA for power factor allocation in the VLC system, Figure 7 shows the BER curves for five agents at different locations. The results consist of a Signal-to-Noise Ratio (SNR) between 0 to 150. The blue curve shows agent-1 at a distance of 6 m inches, which is the farthest agent in the NOMA setup. The high power factor is allocated to this user. The orange curve shows the BER rate for agent-2 at a distance of 4.5 m in an indoor environment. The yellow curve shows the BER for agent-3 at a distance of 3.8 m. The purple and green curves show the BER curves for agent-4 and agent-5 at distances of 2 and 0.7 m, respectively. Agent-5 has been allocated low power based on the distance. As expected, the BER for the high-power agent shows sharp and quick decay; however, the low-power agent shows slow and high BER values. The higher BER shows the signal suffers higher noise during signal propagation from the base station to the agent. At a given time instance, the power allocation is performed based on the location of the agent. However, the value of the power factor based on distance from the base station is already stored in a look table using the simplified gain ratio power allocation (S-GRPA) method. The power factor for each agent is allocated based on the location found by the RFR algorithm. It can be seen that S-GRPA with RFR is able to allocate appropriate power factors to each agent based on the location in an indoor environment.

## 4. Conclusions

Indoor localization and task accomplishment depend on the communication link between the base station and mobile sensor networks, such as multi-agent systems for collaborative tasks that involve ground mobile robots and drones. In this research work, a power allocation method using the precise location of the agent is proposed for the power-domain non-orthogonal multiple access (P-NOMA). P-NOMA enables the base station to suppress signals for different agents using the same time-frequency channel, and it requires the information of the channel for better power allocation to each agent. The channel is highly affected by the distance between the end agent and the base station. In this research work, a machine learning algorithm is proposed to find the location of the agent using Received Signal Strength (RSS) and Angle of Arrival (AoA). The location provided by the machine learning algorithm is used to determine the channel gain and the appropriate power factor is allocated to each agent based on simplified gain ratio power allocation (S-GRPA). It is shown by simulations that Random Forest Regression (RFR) performed better to obtain the location as compared to Support Vector Machine (SVM). In addition, the Euclidian Distance matrix is used to find the location of the agent, if the signal is not received from a particular agent, based on the mutual distances of agents in the network. The complete framework is tested for five agents using the VLC channel. The S-GRPA with the RFR algorithm was able to assign the appropriate power factors to each user based on its location. This work provides bases for dynamic power allocation for multiuser VLC systems. The future work aims to test this method in real-world experiments.

## Figures and Tables

**Table 1:** Simulation parameters.

Parameters	Value
Dimension	6.3 × 2.5 × 2 m^3^
Tx-Rx (LED arrays-PDs on agents) Configuration	4 × 4
No. of LED Arrays	4
No. of LEDs in single Array	60
Total Power	20 watts
Semi-angle at half power	70 degrees
PD field of view	60 degrees
Refractive Index	1.5
No. of Agents	5

**Table 2:** Performance comparison.

Algorithm/Study	Accuracy
Proposed RFR method	0.19 m
Proposed SVM method	0.3 m
Epsilon [12]	4 m
Plugo [54]	0.33 m
WiDeep [55]	1.21 m
Luxapose [13]	0.1 m

## Data Availability

The data presented in this study are available on request from the corresponding author. The data are not publicly available due to privacy and ethical reasons.
